# Phagocytic Activity, Oxygen Metabolism and Serum Amyloid a Concentration in Peripheral Blood of Mink with Subclinical Aleutian Virus Infection

**DOI:** 10.3390/ani12080987

**Published:** 2022-04-11

**Authors:** Andrzej Żmuda, Urszula Lisiecka, Katarzyna Dudek, Roman Dąbrowski, Bolesław Gąsiorek, Stanisław Winiarczyk, Krzysztof Kostro

**Affiliations:** 1 Department of Epizootiology and Clinic of Infectious Diseases, Faculty of Veterinary Medicine, University of Life Sciences in Lublin, Głęboka 30, 20-612 Lublin, Poland; vetgrh@gmail.com (B.G.); genp53@interia.pl (S.W.); kkostro@wp.pl (K.K.); 2 Department of Cattle and Sheep Diseases, National Veterinary Research Institute, Aleja Partyzantów 57, 24-100 Pulawy, Poland; katarzyna.dudek@piwet.pulawy.pl; 3 Department and Clinic of Animal Reproduction, Faculty of Veterinary Medicine, University of Life Sciences in Lublin, Głęboka 30, 20-612 Lublin, Poland; roman.dabrowski@up.lublin.pl

**Keywords:** phagocytosis, oxidative burst, Aleutian disease (AD), serum amyloid A (SAA)

## Abstract

**Simple Summary:**

Aleutian disease (AD) is a chronic disease of mink caused by the Aleutian Mink Disease Virus (AMDV) that results in dysfunction of the immune system. The prevalence of asymptomatic AMDV infections suggests a necessity to explore their effects on the cellular mechanisms of non-specific immunity in farmed mink. In the present study, the phagocytic activity and oxygen metabolism of peripheral blood granulocytes and monocytes in mink with chronic subclinical AMDV infection was assessed. Serum amyloid A (SAA) concentrations were investigated to measure the intensity of inflammatory processes. There was a significant decrease in the phagocytic activity and oxygen metabolism of granulocytes and monocytes in the AMDV-infected mink. SAA value was significantly higher in the group of infected minks compared to the control group. The significant decline in the mean values of the phagocytic activity and oxygen metabolism indicators observed in the minks with chronic AMDV infection, compared with the values noted in the healthy animals, may be a result of immunosuppression of non-specific cellular immunity. Monitoring of serum SAA levels in mink with asymptomatic inflammation may help assess the health of mink and detect asymptomatic inflammation caused by AMDV infection.

**Abstract:**

Aleutian disease (AD) is a chronic disease of mink caused by the Aleutian Mink Disease Virus (AMDV) that results in dysfunction of the immune system. The prevalence of asymptomatic AMDV infections suggests a necessity to explore their effects on the cellular mechanisms of non-specific immunity in farmed mink. The study evaluated the phagocytic activity and oxygen metabolism of peripheral blood granulocytes and monocytes in mink with chronic subclinical AMDV infection. Moreover, the intensity of inflammatory processes was assessed based on the serum amyloid A (SAA) concentration. The analyses involved 24 brown mink females aged 12–24 months. The experimental group (group I) consisted of mink with chronic subclinical AMDV infections, and the control group (group II) included healthy animals. The statistical analysis was performed using the Mann-Whitney U rank test. Phagocytic activity of granulocytes and monocytes was carried out using flow cytometry, and SAA concentration was determined with enzyme-linked immunosorbent assay (ELISA). Compared with the control group, there was a significant decrease in the phagocytic activity and oxygen metabolism of granulocytes and monocytes in the AMDV-infected mink (*p* < 0.05). Additionally, it was found that the mean SAA value was significantly higher in the group infected with AMDV than in the control group (*p* < 0.05). The obtained data indicate that monitoring the serum SAA levels in mink with asymptomatic inflammation may help assess the health of mink and detect asymptomatic inflammation caused by AMDV infection.

## 1. Introduction

Aleutian disease (AD) is a chronic disease of mink caused by the Aleutian Mink Disease Virus (AMDV), the genome of which is composed of single-stranded DNA with a length of ca. 5000 bp. Low production performance on mink farms with endemic AD is caused by chronic AMDV infection [1,2,3]. The intensive and continuous replication of AMDV in the organisms of infected minks results in functional disturbances of the immune system (excessive synthesis of specific antibodies, intense proliferation of plasma cells, and formation of immunological complexes). Aleutian disease in adult mink reared on Polish farms is usually asymptomatic [2]. The increasing problem of the prevalence of asymptomatic AMDV infections suggests a necessity to explore their effects on the cellular mechanisms of non-specific immunity in farmed mink.

Aleutian disease is characterized by continuous and over-stimulation of the humoral immune response leading to plasmacytosis and gammopathy. The presence of antiviral antibodies causes the formation of immune complexes which, by accumulating in the blood vessels of the kidneys, liver, and spleen, cause the inflammation of these organs. Strong suppression of the immune system caused by persistent AMDV infection increases the susceptibility of mink to secondary bacterial infections, which are often the direct cause of numerous deaths, especially of young ones [4].

Aleksandersen et al. [5], using the method of in situ hybridization, showed that B cells are the main site of virus multiplication among the entire population of lymphocytes. The primary role of B cells as target cells for AMDV replication was also confirmed by Aasted and Leslie [6], indicating the possibility of induction of B lymphocytes pseudotransformation under the influence of specific antigenic stimulation. This finding confirmed the dominant role of B cells in viral replication. However, it should be emphasized, that Kanno et al. [7,8,9] and Mori et al. [10], investigated the expression of AMDV nucleic acids in macrophages of lymph nodes in peritoneal exudate and follicular cells of experimentally infected mink.They revealed that 20–30% of the phagocytic cells contained DNA, and 1–2% of the cells contained AMD mRNA and structural proteins. Transcription of viral genetic material in macrophages indicates that they may also be targets for AMDV.

There are indications that Aleutian disease that develops in mink organisms could lead to impaired phagocytosis [11]. The authors claim that mononuclear phagocytic system (MPS) activity in mink in the early stage of the disease is comparable to healthy mink. It is suggested that the MPS blockade in the later stages of the disease may be responsible for some pathologic changes in AD. To the best of our knowledge, there are no more articles concerning this subject. That is why it seems important to investigate this aspect of the immune response during AD in mink.

The study aimed to evaluate the phagocytic activity and oxygen metabolism of peripheral blood granulocytes and monocytes in mink with chronic subclinical AMDV infection. In addition, the intensity of inflammatory processes was assessed based on serum amyloid A (SAA) concentration which is considered one of the acute phase proteins in mink.

## 2. Materials and Methods

### 2.1. Animals and Study Design

The investigations were carried out on two mink farms comprising 45,000 and 25,000 females in the basic herd. The first herd, with 45,000 mink females, was the experimental group. Approximately 60% of the mink from the basic herd reared on this farm were diagnosed with chronic subclinical AMDV infection over three rearing seasons. The AMDV infection was excluded on the other farm comprising 25,000 females, and these animals constituted the control group. The minks from both farms were vaccinated against distemper, viral enteritis, botulism, and *Pseudomonas aeruginosa* infections. The sanitary status on the farms was assessed as good. The animals in both experimental groups were kept in similar conditions. They were fed by the standards recommended for this species with mineral and vitamin supplementation as well as constant access to water. The mink on both farms received identical feeding schemes from the same feed kitchen.

The analyses involved 24 brown mink females aged 12–24 months (Table 1). The bodyweight of the animals is presented in Table 1. They were allocated to the group I and group II with 12 animals in each group. The experimental group (group I) comprised mink from the farm where chronic AMDV infections were diagnosed. The selection of the experimental animals was based on the presence of specific anti-AMDV antibodies detected with the use of the countercurrent immunoelectrophoresis (CIEP) method. Additionally, the presence of viral genomic DNA in blood was confirmed with PCR (Polymerase Chain Reaction). The control group (group II) comprised 12 mink from the AMDV unaffected farm. The animals represented the same breed and were at a similar age as the mink from the group I. In the experimental and control groups, the phagocytic activity and oxygen metabolism of granulocytes and monocytes were determined once during the designed slaughter period (the second half of November) using the flow cytometry method. The investigations received the consent from the II Local Ethics Committee for Experiments on Animals, University of Life Sciences in Lublin (Resolution 30/2010).

### 2.2. Serological Diagnostics of AMDV

Blood samples collected to capillaries intravitally by incision of claws of the females from groups I and II were the biological material for the AMDV serological diagnostics. The analyses were carried out using the countercurrent immunoelectrophoresis method (CIEP), which is routinely used for the diagnosis of Aleutian disease in farmed mink.

### 2.3. Molecular Diagnostics of AMDV

The biological material for the genetic tests included blood samples collected from incised claws of mink, in which specific anti-AMDV antibodies were detected with the use of CIEP. Blood was collected into test tubes containing 5 mM EDTA as an anticoagulant agent. Genomic DNA of the virus was isolated with the use of a DNA isolation kit (DNA Isolation Kit for Mammalian Blood, Roche, Grenzach-Wyhlen, Germany) following the manufacturer’s instructions. The isolated DNA was subjected to amplification with the PCR method, using primers designed based on the AMD virus nucleotide sequences available in the GenBank database (NCBI). The NS1 gene was mainly analyzed, given the large representation of this gene in the database (76). To this end, PCR products that were localized on the gel as predicted during electrophoresis after comparison to the standard (DNA ladder) were sequenced and compared with the nucleotide sequences available in the GenBank database. The analysis resulted in typing the most optimal (conservative) fragments of the AMDV genome as a site of annealing of amplification primers. Before the amplification, the reaction conditions were optimized by experimental determination of the most favorable concentration of polymerase, magnesium ions, deoxynucleotides (dNTP), and primers as well as the hybridization temperature and the number of cycles. Next, the multiplex PCR method was compared with the semi-nested PCR technique in terms of their sensitivity and specificity in the diagnosis of Aleutian disease. The semi-nested PCR method proved to be more specific than the multiplex method with the use of the selected primers.

### 2.4. Polymerase Chain Reaction (PCR)

PCR was carried out using a pair of primers for the NS1 gene. The sequence of primers was: 1F 5′-GGTT-GCTTTACTCCAGAAGA-3′, 1R 5′-ATCACCACCTCCAG-3′, and 2R 5′-TAC-CACACTCTTCAGCCC-3′ from the AMDV genome. This non-structural gene is characterized by the presence of low-variability regions; hence, the primers used for PCR amplification were selected from these regions with the use of the BLAST program available on the NCBI website. The amplification reactions were run at the following temperature profile: 94 °C, 5 min—initial denaturation of the matrix followed by 35 amplification cycles with the following steps: 94 °C 1 min—denaturation, 58 °C 1 min—primer annealing, and 72 °C 1 min—DNA chain extension. The final DNA chain extension was carried out at 72 °C for 5 min. The reaction mixture contained 500 ng of genomic DNA, 2.5 μL 10× of concentrated PCR buffer (100 μM Tris-HCl, pH 8.8, 500 μM KCl), 1.5 mM MgCl_2_, 50 μM dNTP, 0.5 μM of each primer, and 0.5 U of Taq polymerase. The amplification reactions were carried out in a volume of 25 μL using a UNO II thermocycler (Biometra, Göttingen, Niedersachsen, Germany).

### 2.5. Determination of the Phagocytic Activity of Granulocytes and Monocytes 

The phagocytic activity of granulocytes and monocytes was determined with the flow cytometry method using a commercial Phagotest kit (Orpegen Pharma, Heidelberg, Germany). The kit contains FITC-labelled *E. coli* opsonized with antibodies and complement components. It facilitates the determination of the percentage of phagocytizing monocytes and granulocytes and their phagocytic activity (the mean fluorescence intensity indicates the number of bacteria engulfed by the granulocyte). The determination procedure followed the manufacturer’s instructions. Briefly, whole heparinized blood, aliquoted into 50 µL samples, was incubated with 5 µL of FITC-labelled *E. coli* for 10 min at 39 °C to stimulate phagocytosis by white blood cells. Control samples with FITC-labelled *E. coli* were incubated at 0 °C to prevent the phagocytosis. All samples were then placed on ice for 15 min to stop phagocytosis, and the quenching solution was added to distinguish between attached and internalized bacteria. Then, the samples were washed and centrifuged at 1500 rpm for 5 min. In the next step, red blood cell lysis and leukocyte fixation were performed. In order to exclude bacteria and cell aggregates samples were incubated with DNA staining solution for 10 min light protected, on ice. After all these procedures, the cell suspension was analyzed in the flow cytometer. Neutrophils and monocytes were gated based on forward and right-angle light scatter (FS and SS) parameters. All samples were analyzed using the same protocol and identical settings.

### 2.6. Determination of Oxygen Metabolism in Granulocytes and Monocytes

The oxygen metabolism of granulocytes was determined with a commercial Bursttest kit (Orpegen Pharma, Heidelberg, Germany). The test procedures were performed according to the instructions of the manufacturer. Opsonized *E. coli* bacteria were used for activation of the cells in the whole blood. Each blood sample was incubated with unlabeled opsonized *Escherichia coli* strain LE392 for 10 min in a water bath at 39 °C. A sample without stimulus served as a negative control. After initial incubation dihydrorhodamine 123 (DHR-123) solution was added to all of the samples and they were incubated at 39 °C for 20 min. Reactive oxygen species (ROS) oxidize non-fluorescent dihydrorhodamine 123 to fluorescent rhodamine 123, and this reaction allows the detection of ROS during the oxidative burst. After 20 min, the incubation was stopped by placing the samples on ice for 15 min and adding a cold lysing solution. Then, after the washing step, the DNA staining solution was added to stain the bacterial nucleic acid and the cells. The percentage of cells producing ROS and mean fluorescence MFI (enzymatic activity) were analyzed. The gating strategy was similar to that in phagocytosis.

### 2.7. Determination of the Serum SAA Protein Level

Serum amyloid A (SAA) concentrations were determined using a commercial ELISA kit (Multispecies SAA ELISA kit; Tridelta Development Ltd., Maynooth, Ireland) according to the manufacturer’s instructions. The absorbance for each standard and sample was detected in an automated plate reader (Elx800 Microplate Reader, BioTek Instruments, Inc., Winooski, VT, USA) at 450 nm using the KC Junior program from the same manufacturer. The working calibration curve (C1–C6) was prepared using serial dilutions of the SAA calibrator provided in the kit. All samples were pre-diluted at 1:500. If the result did not meet the calibration curve, the samples were further diluted and re-analyzed. The concentration of each sample was determined from the calibration curve and then multiplied by the appropriate dilution factor. 

### 2.8. Statistical Analysis

The results were analysed in the Statistica 10.0 program. The significance of differences was calculated using the Mann-Whitney U rank test at *p* < 0.05. The statistical mean and the standard error of the mean were calculated for all the results.

## 3. Results

**Figure 1:**
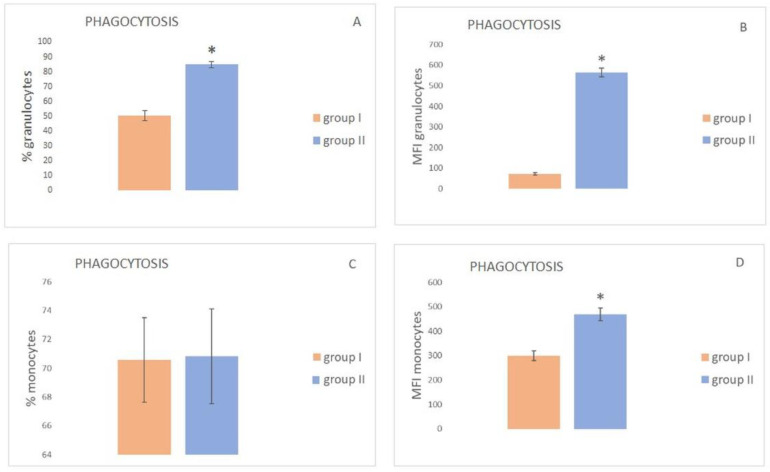
Cytometric analysis of the phagocytic activity of granulocytes and monocytes in the peripheral blood of mink with subclinical AMDV infection and the control group (x ± SE). (**A**) % of granulocytes performing phagocytosis; (**B**) MFI of granulocytes performing phagocytosis; (**C**) % of monocytes performing phagocytosis; (**D**) MFI of monocytes performing phagocytosis. Group I—mink with subclinical AMDV infection, Group II—mink without subclinical AMDV infection, *—statistically significant difference between the groups at *p* < 0.05.

Cytometric analysis of the phagocytic activity and oxygen metabolism in granulocytes and monocytes The results of the assessment of the phagocytic activity of granulocytes and monocytes in peripheral blood of the experimental and control mink are shown in Figure 1. The mean percentage of phagocytizing granulocytes in group I was significantly lower than in group II, with mean values of 50.5 ± 3.2 and 84.8 ± 2.8, respectively (Figure 1A). The mean fluorescence intensity MFI exhibited a similar trend (Figure 1B). The MFI value in group I was 71.9 ± 6.29, which was significantly lower than in the case of the control animals, i.e., 565.2 ± 21.32 (Table 2).

The results of the evaluation of the phagocytic activity of monocytes of the group I and II mink are presented in Figure 1C,D. The data shown in Figure 1C does not reveal a statistically significant (*p* < 0.05) difference in the percentage of phagocytizing cells in the mink from group I, in comparison with the control group. However, the MFI values differed significantly between the groups (Figure 1D).

**Figure 2:**
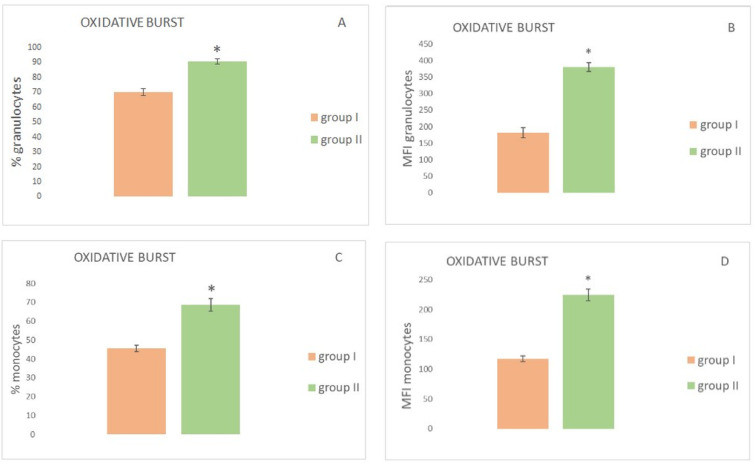
Oxygen metabolism in granulocytes and monocytes in the peripheral blood after PMA activation in mink with subclinical AMDV infection and the control group (x ± SE). (**A**) % of granulocytes performing oxidative burst; (**B**) MFI of granulocytes performing oxidative burst; (**C**) % of monocytes performing oxidative burst; (**D**) MFI of monocytes performing oxidative burst. Group I—mink with subclinical AMDV infection, Group II—mink without subclinical AMDV infection, *—statistically significant difference between the group at *p* < 0.05.

The results of the determination of the metabolic activity of granulocytes and monocytes in the AMDV infected mink and the control group are shown in Figure 2. The data presented in Figure 2A,B reveals that both the percentage of activated granulocytes and the mean fluorescence intensity in the group of the asymptomatic AMDV mink were significantly lower than in the control group.

Similarly, both the percentage of activated monocytes and the mean fluorescence intensity were significantly lower in group I than in group II, (Figure 2C,D), i.e., 45.6 ± 1.5 and 117.2 ± 4.8 (group I) as well as 68.6 ± 3.3 and 224.36 ± 9.76 group II), respectively (Table 2).

**Figure 3:**
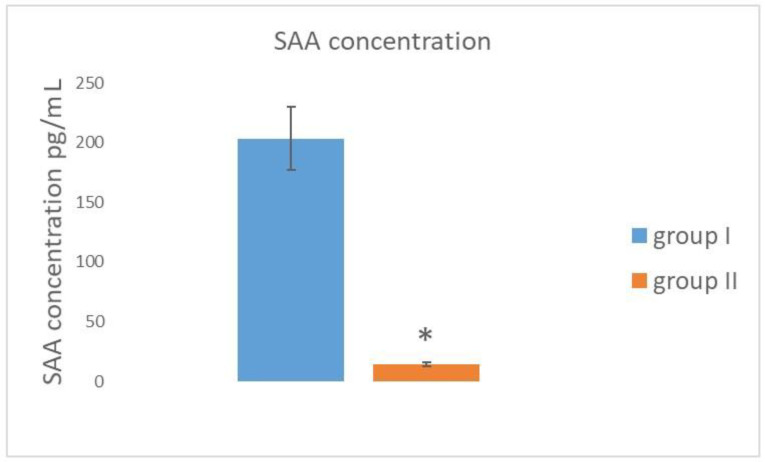
Mean values of the serum SAA level in the peripheral blood of mink with subclinical AMDV and the control group (x ± SE). Group I—mink with subclinical AMDV infection, Group II—mink without subclinical AMDV infection, *—statistically significant difference between the groups at *p* < 0.05.

The data presented in Figure 3 indicates that the concentration of SAA in the peripheral blood of the AMDV-infected mink (group I) was significantly increased compared to the healthy mink (group II). The mean SAA concentration was 203.2 ± 26.43 pg/mL in group I and 14.2 ± 1.4 pg/mL in the control group.

## 4. Discussion

In the present study, granulocytes and monocytes of the mink with subclinical AMDV infection and the control animals were investigated using flow cytometry. Analysis of the phagocytic activity of granulocytes and monocytes (percentage of phagocytizing cells. mean fluorescence intensity) in the peripheral blood of the mink with subclinical AD and the AD-unaffected animals revealed that the mean values of the parameters strictly depended on the health status of the animals. A similar situation was observed concerning the oxygen metabolism of granulocytes and monocytes (percentage of activated cells, mean fluorescence intensity) in these animals. Compared with the control group, there was a significant decrease in the phagocytic activity and oxygen metabolism of granulocytes and monocytes in the AMDV-infected mink (group I). This was particularly evident in the mean fluorescence intensity (MFI), which is an indicator of the intensity of the phagocytosis process and the oxygen-dependent antimicrobial activity of granulocytes and monocytes. It can be assumed that the decline in the phagocytic activity and oxygen metabolism observed in the granulocytes and monocytes is associated with the chronic AMDV infection and its suppressive effects on the cellular mechanisms of non-specific immunity [12,13]. It is widely believed that an efficient phagocytic cell system is one of the key mechanisms of anti-infectious immunity. During infection, neutrophils are the predominant population of phagocytic cells, which constitute a rapid response mechanism by migration from peripheral blood into the inflammation site [14,15]. Disturbances in the biological function of granulocytes and monocytes in mink with natural AMDV infection probably result in the incapability of complete elimination of the infection. Suppressing of non-specific immunity results in a blockade of surface receptors, the inhibition of intracellular metabolism, chemotaxis, and adhesion of phagocytic cells, enhanced production of pro-inflammatory cytokines, and increased levels of selected acute-phase proteins [16]. The concentration of these acute-phase proteins also reflects the level of activation of the immune system during inflammation [17,18,19]. The present study has demonstrated that the SAA protein, which is the main acute-phase protein in mink, can be used as an indicator in this animal species, as it is a sensitive biochemical inflammation marker [20]. As there are no reports on both the reference values and the suitability of SAA determination in mink as an indicator of the intensity of AMDV inflammation processes, the interpretation of the results obtained in the present study is impeded. Our study revealed that the mean SAA value was significantly higher in group I (infected with AMDV) than in the control group (Figure 3). The initiation of enhanced SAA production is triggered by proinflammatory cytokines, in particular IL-1, which is one of the main inducers of increased SAA synthesis in animals [21]. Therefore, the serum SAA level, reflecting the state of activation of the immune system, can be a biochemical marker in the assessment of farmed mink health, especially in mink with asymptomatic AMDV infection. Additionally, the data obtained in the present study imply that monitoring the serum SAA level in mink with asymptomatic inflammation can be helpful in the evaluation of the mink health status in the period preceding specific immunoprophylaxis and in the analysis of the effectiveness of the vaccine used. The increase in the SAA level may have resulted from the enhanced production of proinflammatory cytokines. The cytokines are the main signals of induction of gene transcription factors involved in changes in the biosynthesis of SAA protein in hepatocytes during the development of inflammation [22]. The significantly higher increased serum SAA level in the mink from group I, compared with the healthy mink, is the result of the long-term persistence of the asymptomatic inflammation caused by the AMDV infection.

## 5. Conclusions

It can therefore be assumed that the significant decline in the mean values of the phagocytic activity and oxygen metabolism indicators observed in the minks with chronic AMDV infection, in comparison with the values noted in the healthy animals, may be a result of immunosuppression of non-specific cellular immunity. The immunosuppression may be induced by the persistent inflammation associated with the chronic viral infection. This allows a concurrent conclusion that the dysfunction of the cellular mechanisms of innate immunity in mink with asymptomatic AMDV infection can be one of the causes of secondary bacterial infections development and the low efficacy of specific bioformulations applied in the immunoprophylaxis of infectious diseases in mink. However, the investigated and control groups of minks were relatively small. Therefore more extensive research should be conducted to confirm these conclusions fully.

## Figures and Tables

**Table 1:** The age and weight of animals included in the study.

Group I			Group II	
No.	Age (Months)	Body Weight (kg)	Age (Months)	Body Weight (kg)
1	24	1.8	12	1.9
2	24	1.9	12	1.8
3	12	1.8	24	2.0
4	12	1.7	24	1.8
5	24	1.9	12	1.7
6	24	1.8	24	1.9
7	12	1.7	12	2.0
8	24	1.9	24	1.7
9	12	1.8	12	1.9
10	12	1.7	12	1.9
11	24	1.8	24	1.8
12	24	1.8	24	2.1

**Table 2:** Descriptive statistics of variables (full sample n = 24).

Descriptive Statistics								
Variable	Health Status	Mean	MED	SD	SE	Min	Max	*p*-Value
SAA concentration	AMDV-infected	203.2	191.7	91.5	26.4	70.3	335.9	<0.00001
	healthy	14.2	13.5	4.6	1.4	8.7	22.4	
% phagocytic granulocytes AMDV	AMDV-infected	50.5	52.7	12.2	3.2	29.6	68.4	<0.00001
	healthy	84.8	83.1	7.2	2.1	74.2	95.6	
% phagocytic monocytes AMDV	AMDV-infected	70.6	72.2	10.2	2.9	48.6	84.2	=0.955206
	healthy	70.8	72.5	11.3	3.3	52.5	89.7	
MFI phagocytic granulocytes AMDV	AMDV-infected	71.9	69.5	21.8	6.3	38.7	121.8	<0.00001
	healthy	565.2	559.2	73.8	21.3	456.8	687.7	
MFI phagocytic monocytes AMDV	AMDV-infected	299.7	313.8	67.9	19.6	159.4	389.6	=0.00003
	healthy	469.3	485.4	89.5	25.8	264	612.8	
% granulocytes performing oxidative burst AMDV minks	AMDV-infected	70.11	71.55	7.77	2.24	56.9	79.2	<0.00001
	healthy	90.6	91.2	6.1	1.7	78.9	98.2	
% monocytes performing oxidative burst AMDV minks	AMDV-infected	45.6	45.4	5.5	1.5	38.1	55.4	<0.00001
	healthy	68.6	70.8	11.3	3.3	54.3	82.8	
MFI granulocytes performing oxidative burst AMDV minks	AMDV-infected	181.7	195.2	52.3	15.1	89.4	252.5	<0.00001
	healthy	379.4	391.9	44.4	12.8	300.1	435.6	
MFI monocytes performing oxidative burst AMDV minks	AMDV-infected	117.2	112.4	16.6	4.8	93.1	144.1	<0.00001
	healthy	224.4	217.2	33.8	9.7	189.6	287.4	

## Data Availability

The raw data supporting the conclusions of this article will be made available by the corresponding author upon reasonable request.
